# Are sedatives and hypnotics associated with increased suicide risk of suicide in the elderly?

**DOI:** 10.1186/1471-2318-9-20

**Published:** 2009-06-04

**Authors:** Anders Carlsten, Margda Waern

**Affiliations:** 1 Social Medicine, Department of Public Health and Community Medicine, Sahlgrenska Academy at Gothenburg University and Nordic School of Public Health, Gothenburg, Sweden; 2 Department of psychiatry and neurochemistry, Sahlgrenska Academy at Gothenburg University, Gothenburg, Sweden

## Abstract

**Background:**

While antidepressant-induced suicidality is a concern in younger age groups, there is mounting evidence that these drugs may reduce suicidality in the elderly. Regarding a possible association between other types of psychoactive drugs and suicide, results are inconclusive. Sedatives and hypnotics are widely prescribed to elderly persons with symptoms of depression, anxiety, and sleep disturbance. The aim of this case-control study was to determine whether specific types of psychoactive drugs were associated with suicide risk in late life, after controlling for appropriate indications.

**Methods:**

The study area included the city of Gothenburg and two adjacent counties (total 65+ population 210 703 at the start of the study). A case controlled study of elderly (65+) suicides was performed and close informants for 85 suicide cases (46 men, 39 women mean age 75 years) were interviewed by a psychiatrist. A population based comparison group (n = 153) was created and interviewed face-to-face. Primary care and psychiatric records were reviewed for both suicide cases and comparison subjects. All available information was used to determine past-month mental disorders in accordance with DSM-IV.

**Results:**

Antidepressants, antipsychotics, sedatives and hypnotics were associated with increased suicide risk in the crude analysis. After adjustment for affective and anxiety disorders neither antidepressants in general nor SSRIs showed an association with suicide. Antipsychotics had no association with suicide after adjustment for psychotic disorders. Sedative treatment was associated with an almost fourteen-fold increase of suicide risk in the crude analyses and remained an independent risk factor for suicide even after adjustment for any DSM-IV disorder. Having a current prescription for a hypnotic was associated with a four-fold increase in suicide risk in the adjusted model.

**Conclusion:**

Sedatives and hypnotics were both associated with increased risk for suicide after adjustment for appropriate indications. Given the extremely high prescription rates, a careful evaluation of the suicide risk should always precede prescribing a sedative or hypnotic to an elderly individual.

## Background

The use of psychotropic drugs among the elderly is high [1-3] and health risks associated with these drugs are a topic of public health concern. Increased risk for fall accidents [4,5], adverse drug reactions [6], drug related morbidity [7] and unfavourable interactions with other medication [8] have been emphasized in the literature. While the induction of suicidality is a concern in younger age groups [9], there is increasing evidence that antidepressants may be beneficial in the prevention of suicide late in life [10-15]. Regarding a possible association between other types of psychoactive drugs (sedatives, hypnotics, antipsychotics) and suicide, results are inconclusive [16-18]. Sedatives and hypnotics are widely prescribed to elderly persons with symptoms of depression, anxiety, and sleep disturbance. They are regularly detected in post-mortem analyses of elderly suicide victims, and often implicated in lethal overdoses in Sweden [19].

**Figure 1:**
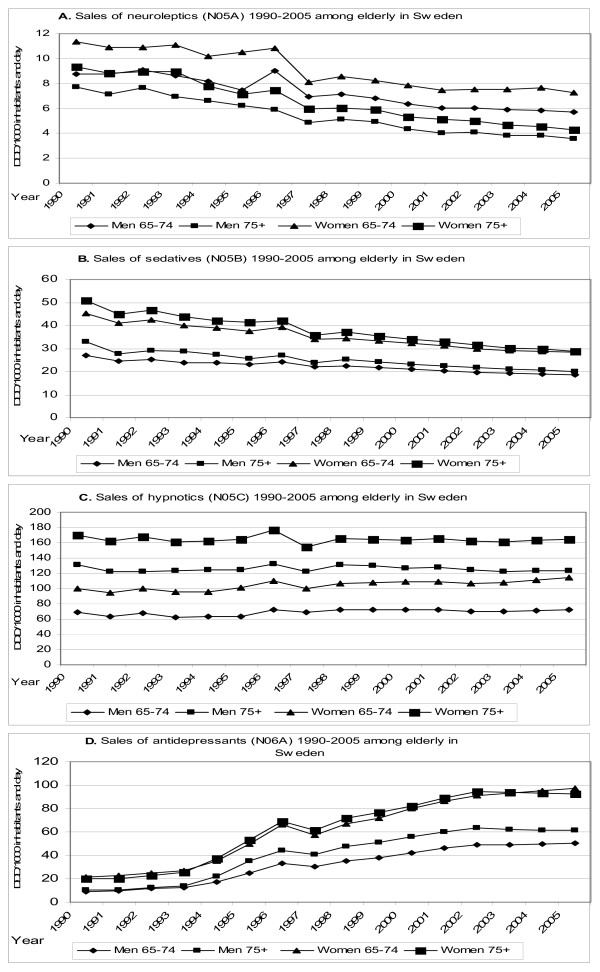
**The sales of psychotropic drugs 1990–2005 among elderly in Sweden**.

The elderly consume more psychotropics than any other age group in Sweden [20]. There have been substantial changes in the sales of psychotropic drugs to persons aged 65 and above over the last fifteen years. While sales of antipsychotics (Figure 1a) and sedatives (Figure 1b) decreased by almost 50% during this time period, sales of hypnotics remained extremely high (Figure 1c). Sales rates are particularly high in the older elderly, with Defined Daily Dosages (DDD)/1000 inhabitants and day as high as 165 in women and 120 in men (Figure 1c). SSRIs were introduced in Sweden in 1990 and antidepressant sales have seen a near five-fold increase since that time (Figure 1d.)

Given the high psychotropic prescription rates and the high suicide rates in this age group it would be appropriate to examine whether different types of psychotropic drugs are associated with increased suicide risk. When testing for a possible association, it is important to rule out confounding by indication. However, studies that include detailed evaluation of psychiatric symptoms in elderly suicides and population-based controls are lacking. The aim of the current study was thus to determine whether different types of psychotropic drugs were associated with increased risk of suicide in persons aged 65 years and above after adjustment for appropriate indications.

## Methods

One hundred consecutive cases of suicide among persons aged 65 years and above who underwent necropsy at the Gothenburg Institute of Forensic Medicine were reviewed. Close informants for 85 suicide cases (46 men, 39 women, mean age 75 years) accepted to participate in an interview with a psychiatrist (MW). We have previously shown that the study cases were representative of all suicides among persons age 65 and above in the catchment area during the study period; antidepressants and/or lithium were detected at post-mortem analysis in 38% of the study cases and 40% of the 100 suicides (65+) evaluated at the Forensic Institute during the study period [21]. The study area included the city of Gothenburg and two adjacent counties (total 65+ population 210 703 at the start of the study).

In order to create a population comparison group, two people with the same sex, year of birth and zip code as each suicide case were randomly selected from the tax register. If a potential comparison person declined participation, another was invited (up to eight per case). In all, 240 people were invited to take part in the study and 153 participated (84 men, 69 women). Comparison subjects were interviewed face-to-face, using the same questionnaire. Primary care and psychiatric records were reviewed for suicide cases and comparison subjects. All available information was used to determine past-month mental disorders in accordance with DSM-IV [21].

Ongoing prescriptions were classified according to the Anatomical Therapeutic Chemical (ATC) classification [22]. For the purpose of this study the following drugs were classified as sedatives: diazepam, alprazolam, buspirone, hydroxizine and dixyrazine. These were classified as hypnotics: flunitrazepam, nitrazepam, zopiclone, zolpidem, oxazepam, levomepromazine, propiomazine and alimemazine. Dixyrazine is a neuroleptic, but here classified as sedative in accordance with its main clinical use. Levomepromazine, a neuroleptic and alimemazin, an antihistamine were both included in the hypnotic group because they are prescribed in Sweden to elderly with sleep disturbance.

### Ethics

The study was approved by the Ethics Committee at the Faculty of Medicine, Gothenburg University.

### Statistics

Crude odds ratios for suicide were calculated for the different classes of psychotropic drugs with bivariate logistic regression. In a second set of analyses, odds ratios were adjusted for age, sex and appropriate indications (see footnote Table 1) using multivariate logistic regression.

**Table 1:** Psychotropic drugs prescribed to elderly suicide cases (n = 85) and population controls (N = 153).

**Type of drug prescribed**	**Cases** **n (%)**	**Controls** **n (%)**	**OR(95% CI)^a^**	**OR(95% CI)^b^**
Any antidepressant	34 (40)	9 (6)	10.7 (4.8–23.8)	0.9 (0.2–3.2)
SSRIs	23 (27)	8 (5)	6.7 (2.9–15.9)	0.8 (0.2–2.9)
Antipsychotics	9 (11)	4 (3)	4.4 (1.3–14.8)	2.7 (0.8–10.1)
Sedatives	31 (36)	6 (4)	14.1 (5.6–35.6)	4.4 (1.3–15.2)
Hypnotics	48 (56)	16 (10)	10.8 (5.4–21.5)	4.2 (1.6–11.0)

Odds ratios and 95% confidence intervals.

a Unadjusted

b Adjusted for age, sex and indication. Antidepressants adjusted for affective and anxiety disorders, antipsychotics adjusted for psychotic disorders, sedatives and hypnotics adjusted for any DSM IV Axis I disorder.

## Results

Psychotropic drugs were widely prescribed to the suicide cases and all drug types were associated with suicide in the unadjusted analyses (Table 1). Antidepressants were prescribed to 40% of the cases at the time of the suicide. Antidepressant medication was associated with a ten-fold increase in suicide risk in the initial analysis. However, after adjustment for affective disorders and anxiety disorders, neither antidepressants in general nor SSRIs in particular showed an association with suicide.

Antipsychotics were prescribed to one tenth of the suicide group at the time of death. Only 3% of those in the comparison group had a prescription for an antipsychotic. Antipsychotic medication was associated with increased suicide risk in the initial analyses, but the association did not remain after adjustment for psychotic disorder.

While sedative use was uncommon among persons in the control group, over a third of the suicide cases were prescribed sedatives at the time of death. A prescription for a sedative was associated with an almost twelve-fold increase of suicide risk after adjustment for anxiety disorders (OR 11.8 C.I. 4.6–30.6). As sedatives may be indicated for anxious elderly with other types of mental disorder (depression, psychosis) a second model was constructed which adjusted for any DSM IV disorder. Sedative treatment remained an independent four-fold risk factor for suicide even in the fully adjusted model (Table 1).

Hypnotics were the most widely prescribed drug type in both cases and controls. Half of the suicide cases had a prescription for a hypnotic at the time of the suicide. Hypnotics were prescribed to one tenth of the comparison group. A prescription for a hypnotic drug was associated with a ten-fold increase in suicide risk in the crude analysis. As sleep disturbance may occur in most psychiatric disorders, the final model was adjusted for any Axis I disorder. Having a current hypnotic prescription was associated with a four-fold increase in suicide risk in this adjusted model.

As interactions between psychoactive drugs and alcohol may trigger impulsive behaviour, we wanted to test whether suicide victims who used sedatives/hypnotics were more likely to have a positive post-mortem test for alcohol. For hypnotic users, 11 out of 47 cases had a positive test for alcohol compared to 14 out of 35 for non-users (p = 0.146, Fisher's exact test). For sedative users, 8 out of 31 had positive registration for alcohol compared to 17 out of 51 for non-users (p = 0.622, Fisher's exact test).

## Discussion

We found a four-fold increased suicide risk among elderly using sedatives and/or hypnotics after adjustment for appropriate indications. A recent Canadian register-based study showed a similar odds ratio [23], but it should be noted that those results pertained specifically to use of benzodiazepines, and diagnoses were based on physicians claims files. Our finding is at odds with that of Barak and colleagues [18] who compared rates of benzodiazepine prescription in elderly patients with major depression who did/did not attempt suicide. The proportion with benzodiazepines was larger in the group that did not attempt suicide. Several methodological differences may help to explain the conflicting results. First, the outcome variable in the study by Barak and colleagues was attempted rather than completed suicide. Second, controls in that study were psychiatric patients; our study employed face-to-face interviews with individuals randomly selected from the underlying population. Third, the proportion of women was greater in the Barak study. Women tend to have higher rates of sedative use than men, and men higher suicide rates than women.

One possible explanation for the observed increase in suicide risk associated with sedatives and hypnotics in our study may be that these drugs trigger aggressive behaviour [24]. Further, interactions between benzodiazepines and alcohol may intensify impulsive tendencies, thereby increasing risk of suicide. We have previously reported that 29% of the suicide cases in the current study had a positive post-mortem test for alcohol [25]. However, proportions a positive post-mortem test for alcohol were similar in suicide cases both with and without sedatives/hypnotics, indicating that interaction with alcohol cannot fully explain the observed increase in risk. Another partial explanation might be that persons with prescriptions for these drugs have ready access to a suicide method. Availability of suicide methods increases suicide risk [26]. The current study design does not allow us to tease out the contribution of availability of suicide means on suicide risk. Suicide methods vary widely in different cultural settings. It would be of interest to test whether sedatives and hypnotics are associated with increased late-life suicide risk even in settings where other suicide methods, such as hanging or shooting are more common.

The finding that sedatives and hypnotics were associated with increased suicide risk does not in itself imply causality. It is possible that these drugs are merely markers for some other factor related to suicide risk, such as somatic illness, functional disability, alcohol use disorder, interpersonal problems, lack of social network [27] and sleep disturbance [28]. Persons with these problems might be more likely to seek health care and perhaps more likely to receive prescriptions for psychotropic drugs.

To the best of our knowledge, this is the first study to examine use of psychotropic drugs in elderly suicides and matched population controls who have been subjected to a detailed evaluation of psychiatric symptoms. We did not find support for the hypothesis that SSRIs increase suicide risk in the elderly, after controlling for indication. Taken together with previous observations from ecological studies [10-13], from retrospective analyses of patient records [18] and register studies [14], this is important information for the clinician, who might be less inclined to prescribe antidepressants in the aftermath of the "black box warning" that was issued regarding risks in adolescents [29].

### Strengths and limitations

A major strength of this study was the inclusion of detailed psychopathological data which eliminated issues of confounding by indication. Study cases were representative of all suicides in the catchment area [21]. Major limitations include small numbers and the fact that diagnoses of the suicide victims are based on data accrued by proxy interviews. However, there is evidence that suicide studies based on proxy interviews with next of kin (so-called "psychological autopsies") provide reliable diagnoses [30]. Further, medical records were readily available for cases and controls, and these were authored by physicians who were "blind" to the suicide outcome. Another methodological concern was that the participation rate for the comparison subjects was lower (64%) than that of the suicide informants (85%). Potential comparison persons with mental illness and psychotropic drug treatment might be less likely to participate, which would result in inflated odds ratios.

### Implications for the clinician

While antidepressant prescription was not an independent predictor of suicide risk, it is noteworthy that 40% of the elderly cases committed suicide despite prescribed treatment for affective illness. Treatment failure cannot be ascribed to lack of adherence to antidepressants, as we have previously shown that antidepressants were detected at post-mortem screening to a large degree [21]. As pointed out by Szanto and colleagues [31], there is a need for intensive treatment and follow-up of the depressed and suicidal elderly. In the present study, sedatives and hypnotics were related to increased risk for late life suicide. Clinicians need to be aware of this as these drugs are widely prescribed to the elderly. A careful evaluation of the suicide risk should be carried out when an elderly person presents with symptoms of anxiety and sleep disturbance.

## Conclusion

In conclusion sedatives and hypnotics were both associated with increased risk for suicide after adjustment for appropriate indications. Given the extremely high prescription rates, a careful evaluation of the suicide risk should always precede prescribing a sedative or hypnotic to an elderly individual.

## Competing interests

The authors declare that they have no competing interests.

## Authors' contributions

MW was responsible for the design of the study and the study interviews. AC was responsible for collection and analysis of the pharmacological data and performed the statistical analyses. Both AC and MW prepared the manuscript. Both authors read and approved the final manuscript.

## Pre-publication history

The pre-publication history for this paper can be accessed here:


